# Supplementary material for: Comparative Genomics Revealing Insights into Niche Separation of the Genus Methylophilus
Source: Microorganisms. 2021 Jul 24;9(8):1577. doi: 10.3390/microorganisms9081577 (PMC8399692; doi:10.3390/microorganisms9081577)
Supplement: Supplementary file 1 [file microorganisms-09-01577-s001.zip › microorganisms-1249801-supplementary/Supplementary Figures.pdf]

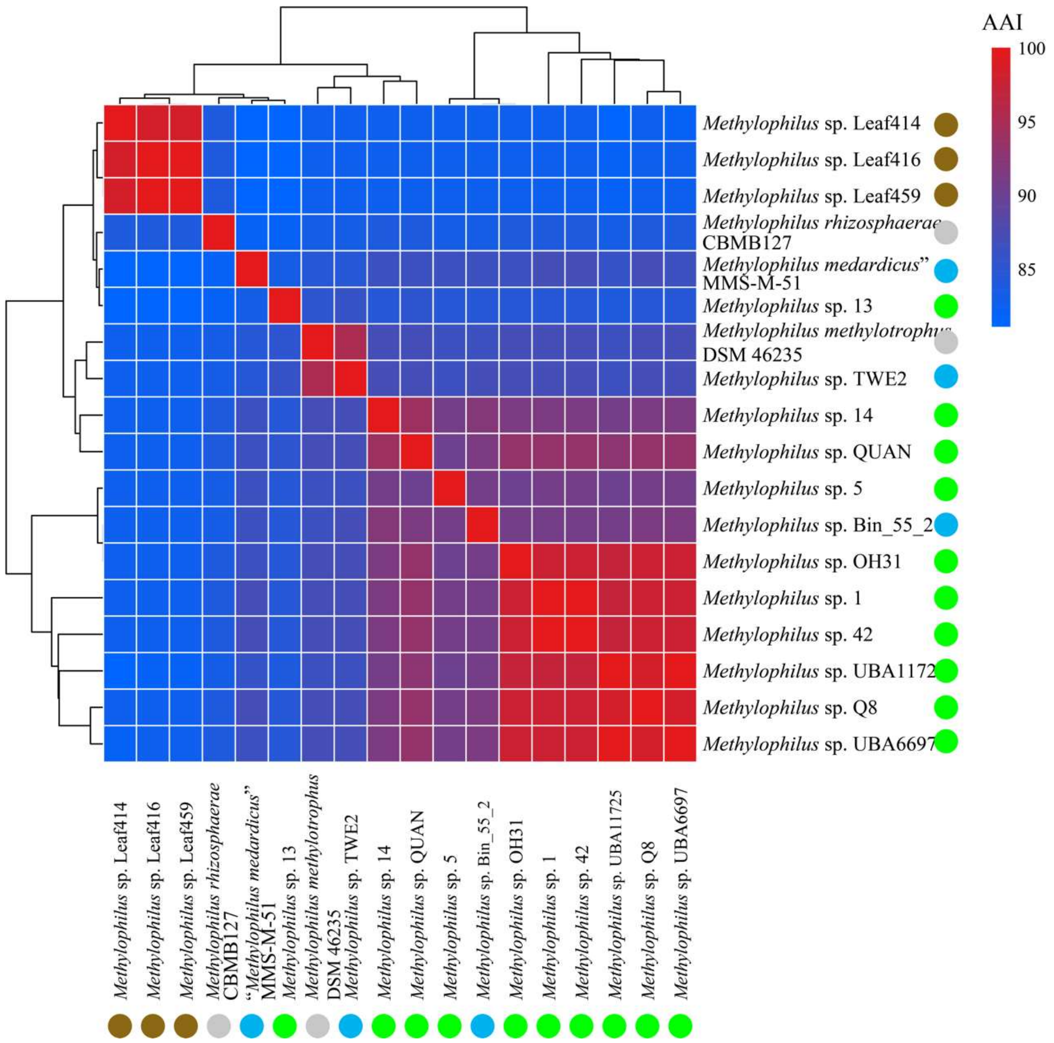

Supplementary Figure S1. AAI heatmap. Cells in the heatmap consistent with different AAI values were represented by different color scales. The solid circles with colors, blue, brown, green and grey, represent isolation sources, water, leaf, sediment and others, respectively.

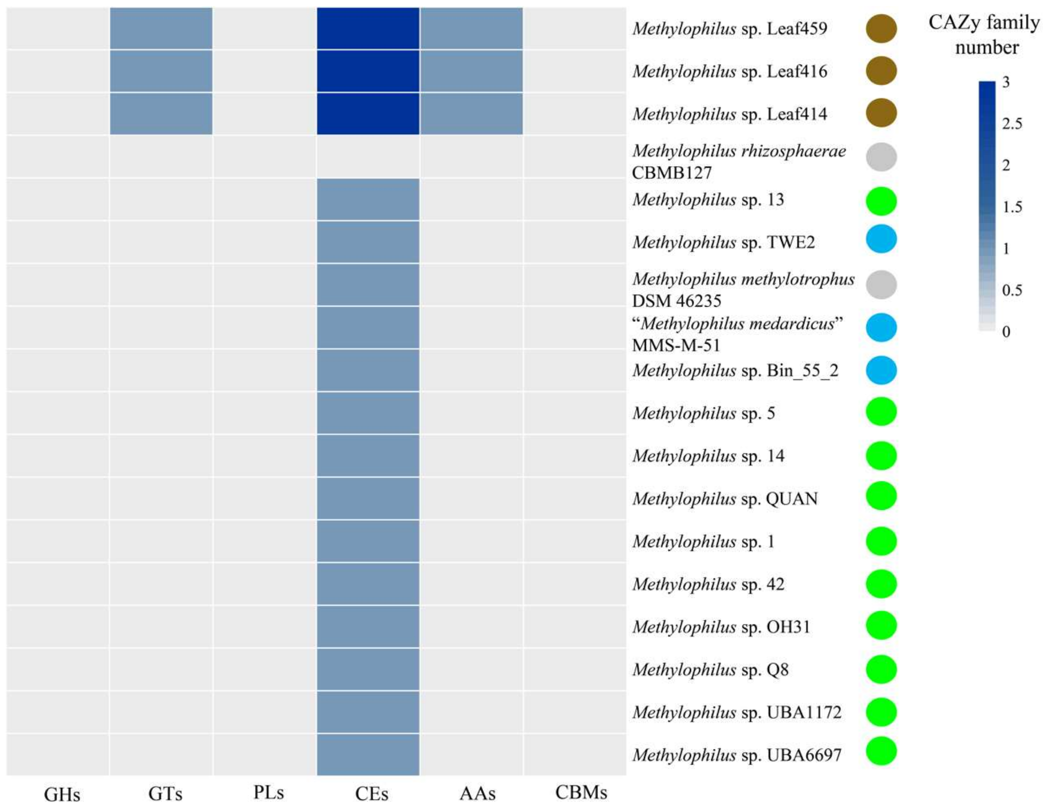

Supplementary Figure S2. Heatmap conducted according to CAZy results. Cells in the heatmap consistent with different CAZy numbers were represented by different color scales. The solid circles with colors, blue, brown, green and grey, represent isolation sources, water, leaf, sediment and others, respectively.
